# Acute Headache Due to Intracerebral Hemorrhage Secondary to Brain Metastases

**DOI:** 10.7759/cureus.16889

**Published:** 2021-08-04

**Authors:** Zachary J Cohen-Neamie, Latha Ganti, Thor S Stead, Joshua Walker, Frank Fraunfelter

**Affiliations:** 1 Emergency Medicine, Trinity Preparatory School, Maitland, USA; 2 Emergency Medicine, Envision Physician Services, Plantation, USA; 3 Emergency Medicine, University of Central Florida College of Medicine, Orlando, USA; 4 Emergency Medicine, Ocala Regional Medical Center, Ocala, USA; 5 Emergency Medicine, HCA Healthcare Graduate Medical Education Consortium Emergency Medicine Residency Program of Greater Orlando, Olrando, USA; 6 Medicine, Warren Alpert Medical School, Providence, USA; 7 Emergency Medicine, University of Central Florida, Orlando, USA; 8 Emergency Medicine, University of Central Florida, Providence, USA

**Keywords:** renal cell carcinoma, brain metastases, intracerebral hemorrhage, headache, abnormal computed tomography

## Abstract

Intracerebral hemorrhage (ICH) is a relatively common condition seen throughout the world, with the vast majority of cases referring to primary ICH. However, secondary ICH from other underlying conditions is also possible. In the present case, the patient presented with severe headaches. An initial computed tomography (CT) was taken which showed hyperdense regions in both the occipital lobe and right lateral ventricle. The patient was hypertensive upon arrival, so medication was given to lower his blood pressure. Due to the patient’s history of hypertension, it was believed to be a case of primary ICH caused by high blood pressure, but because of the odd positioning of the hemorrhaging, it was recommended for magnetic resonance imaging (MRI) and angiography (MRA) to be taken. Using the MRI and MRA, it was found out that growing nodes were responsible for the hypodense regions on the CT. Considering the patient's history of renal cell carcinoma metastasizing to the abdomen and lungs, the nodes were diagnosed as brain metastasis (BM) developed from the patient’s past kidney cancer. Considering the hemorrhaging locations in the brain, it was concluded that the ICH was secondary to BM. After consulting neurosurgery and hematology, the patient was discharged to his family. Although not very prevalent in cases of ICH, BM is a cause that can not be overlooked. Sometimes initial imaging does not reveal such an underlying source. It is always important to pay close attention to the characteristics of the ICH so that it is possible to determine the true reason for the hemorrhage.

## Introduction

Intracerebral hemorrhage (ICH) is any type of bleeding that occurs in the brain tissue. More than 1 million people around the world are diagnosed with ICH annually [1]. The most common cause of ICH is hypertension, due to cerebrovascular damage to small arteries and arterioles [2]. Other causes include arteriovenous malformations, amyloid angiopathy, and trauma [3]. Hemorrhage associated with brain metastases (BM) is an uncommon cause of ICH. They can either appear as the presenting symptom of BM, or can be found after the BM was already discovered [4]. Hemorrhage resulting from brain tumors can occur in up to 10% of all primary metastatic tumors [2]. ICH is also associated with secondary metastatic brain tumors, and accounts for 2%-7% of all spontaneous development of ICH [5,6]. The authors present the case of a man with a history of clear cell renal carcinoma who complained of a severe temporal headache and was found to have ICH secondary to an occipital lobe mass.

## Case presentation

A 57-year-old right-handed male presented to the emergency department (ED) with acute headache. He began having severe headaches, dizziness, nausea, neck pain, and binocular blurry vision, and photophobia a few days prior. The headache was located in the right temporal region, and upon standing or moving his head to the right, the patient experienced dizziness. His past medical history included hypertension, hyperlipidemia, and clear cell renal carcinoma with metastasis to the lungs and abdomen. His past surgical history included a nephrectomy for his stage 2 renal cell carcinoma. The patient reported he drank six beers daily and was a former smoker. The last time the patient drank was five days prior. The patient denied ever taking any illicit drugs.

His initial vital signs included a blood pressure of 172/99 mmHg, a temperature of 97.2º F, a pulse of 86 beats per minute, an oxygen saturation of 99% on room air, and a respiratory rate of 18 breaths per minute. 

Physical examination revealed a well-nourished and well-developed male, who appeared his stated age. His body-mass-index was 32.4 kg/m2. The patient did not have any acute respiratory distress. The patient was awake, alert, and oriented to person, place, time, and situation. His speech was clear. His pupils were equal, round, and reactive to light. Extraocular eye movements were intact, although the patient did have blurry vision in the right lower quadrant of both eyes. His face was symmetrical, and the tongue was midline with no fasciculations or trauma. Sensation was intact to light touch and pinprick. His coordination exam revealed normal finger-nose-finger testing and normal heel-to-shin testing. Patient had 5/5 motor strength of his bilateral upper and lower extremities. His National Institutes if Health Stroke Scale (NIHSS) score was 0. 

His laboratory evaluation showed slight anemia with a hemoglobin of 12.5 g/dL, an elevated sedimentation rate of 100 mm/hr. All other laboratory evaluation was within normal limits.

**Figure 1:**
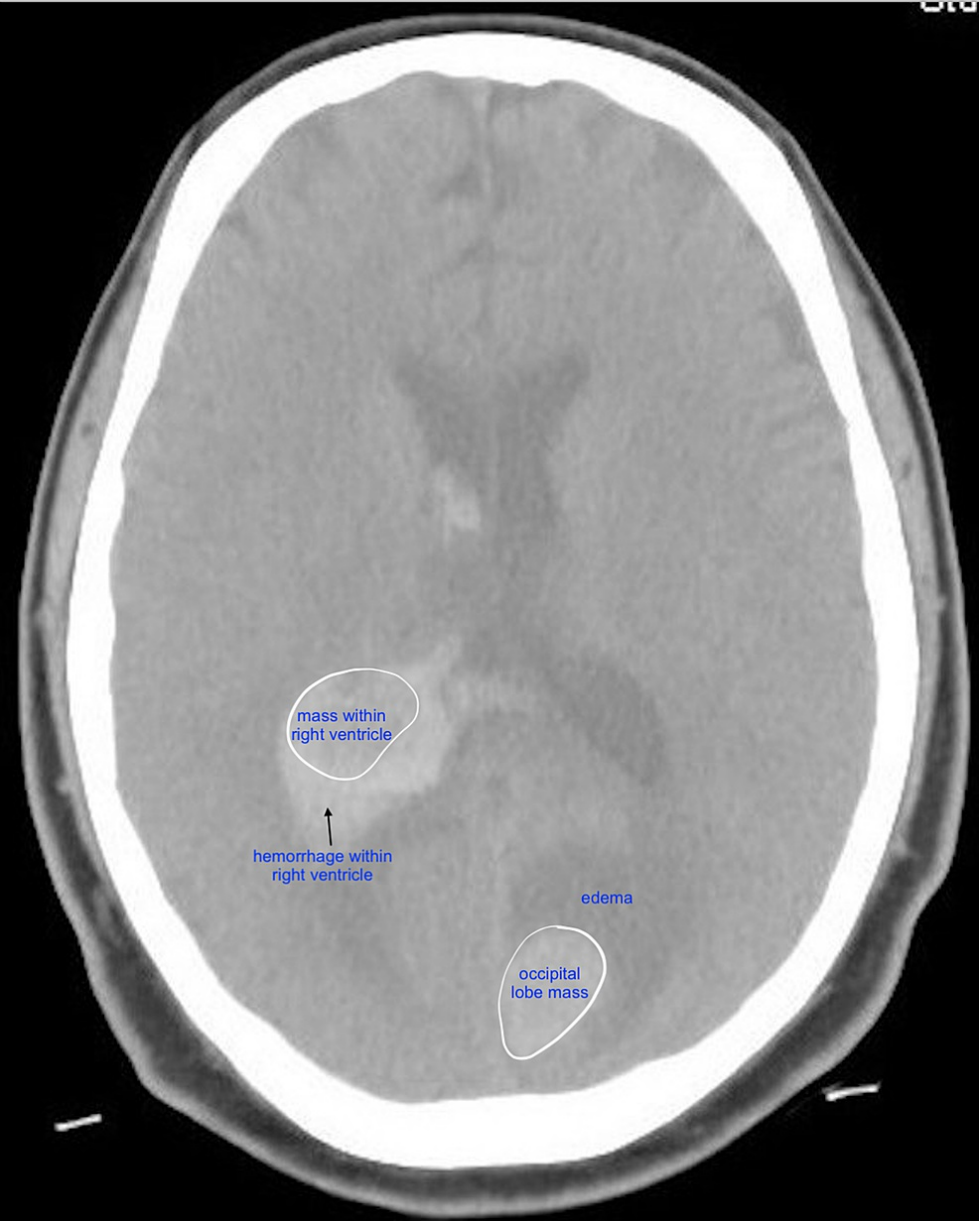
Non-contrast computed tomography (CT) scan demonstrating a 2 cm focal hyperdensity in the medial left occipital lobe, with appearance of intraparenchymal hemorrhage but suspicious for an underlying mass given the surrounding edema.

Non-contrast computed tomography (CT) scan of the brain demonstrated moderate hemorrhage within the right ventricle. A 2.1 cm focal hypodensity in the left medial occipital knob was noted as was a mass within the posterior right ventricle at the area of the hemorrhage (Figure 1).

**Figure 2:**
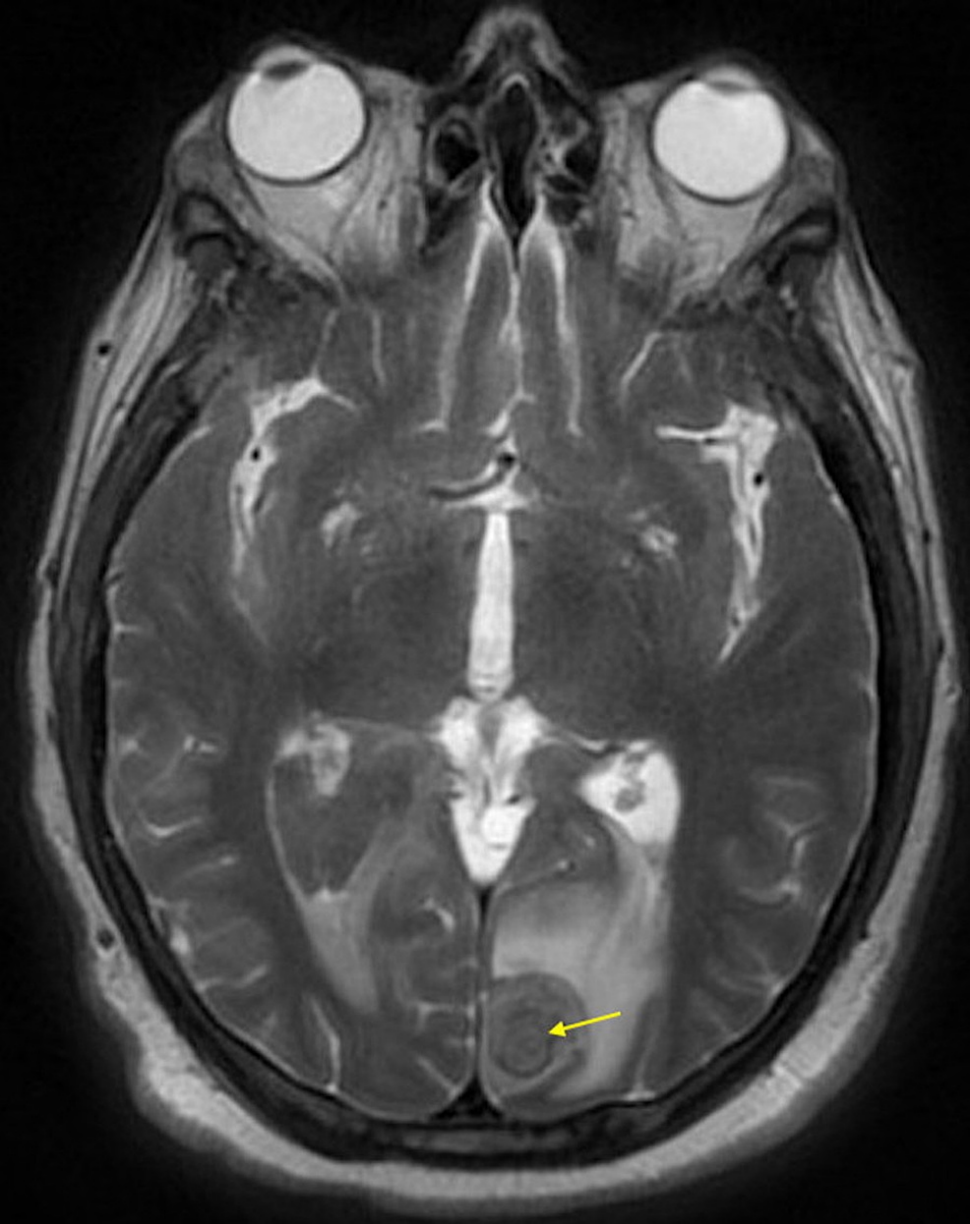
Brain magnetic resonance imaging (MRI) demonstrating focus of acute parenchymal hemorrhage in the left occipital lobe measuring 2.4 x 1.8 x 1.8 cm and an enhancing nodule in that region measuring 1.1 x 1.1 cm (arrow).

Considering the presence of edema surrounding the hyperdense regions, magnetic resonance imaging (MRI) and angiography (MRA) were ordered. MRA of the head was unremarkable. The MRI demonstrated an enhancing nodule in the left occipital lobe with associated parenchymal hemorrhage and surrounding edema and an enhancing nodule in the right cord plexus also with associated right intraventricular hemorrhage and surrounding parenchymal edema, likely due to metastatic disease (Figure 2).

**Figure 3:**
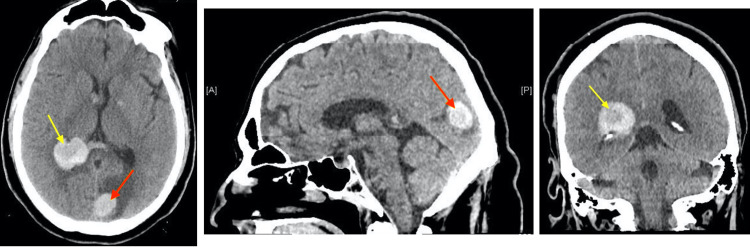
Axial, sagittal, and coronal computed tomography (CT) views demonstrating a 2 cm hyperdense lesion in the medial aspect of the left occipital lobe with moderate surrounding vasogenic edema (red arrow) and right lateral ventricular hemorrhage extending into the occipital and temporal horns (yellow arrow).

The patient was given intravenous dexamethasone for the edema associated with the occipital lobe tumor, and nicardipine infusion for blood pressure control. Repeat CT scan the following day revealed stable appearance of the right lateral ventricular hemorrhage extending into the occipital and temporal horns; and stable appearance of the 2 cm focal hyperdensity in the medial aspect of the left occipital lobe with moderate vasogenic edema (Figure 3).

After being evaluated by neurology, neurosurgery, and hematology, his headache improved and given the paucity of his neurologic impairment, the patient was discharged home to the care of his family.

## Discussion

The differential diagnosis of ICH from metastasis versus primary ICH can be challenging [7-9]. Clues to the former include atypical location of the ICH, non-hemorrhagic tissue within the ICH, multiple sites of ICH, and an uneven distribution of density within the ICH [10]. Edema surrounding the ICH is suspicious for an underlying cerebral metastasis, as is an indentation of the hematoma’s surface [11].

**Figure 4:**
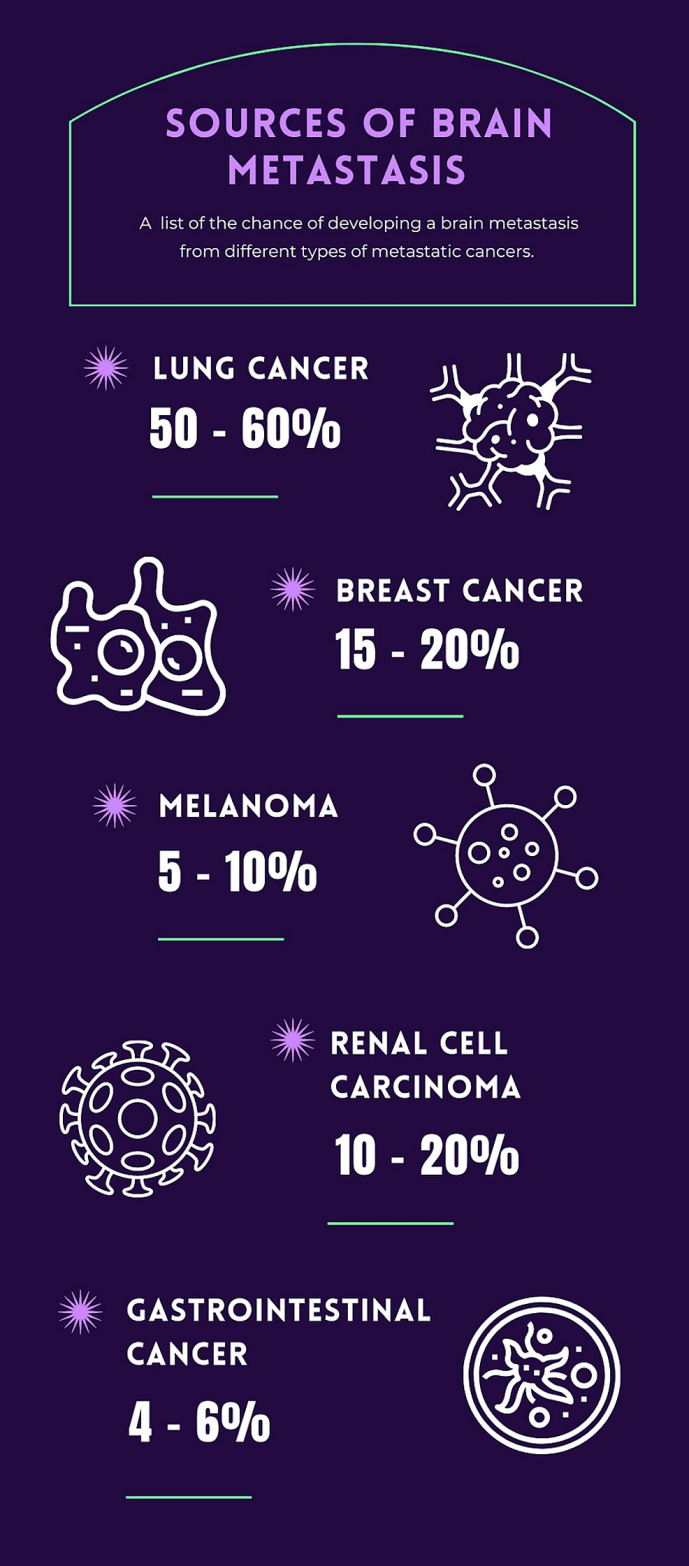
Infographic summarizing sources of brain metastases.

In our patient, initially, the ICH was thought to be due to hypertension, as the patient had a history of uncontrolled blood pressure. However, the ICH was in two locations, which is unusual [12]. It was seen in the lateral ventricle as well as in the occipital region and was a sizable volume. If this ICH were due to a vascular cause, one would expect the neurologic findings to be quite dramatic; however, the patient’s neurological exam was remarkably benign with an NIHSS score of 0. Furthermore, the edema surrounding the area of hemorrhage was also suspicious for ICH of a non-vascular origin. The MRI elucidated the culprit to be a brain mass. BM are the most frequent cerebral tumors in adults with approximately 80% of the metastases located in the cerebral hemispheres and 20% in the posterior fossa structures [13] (Figure 4).

In our patient, the primary cancer was renal cell carcinoma, and his headache presentation revealed the metastases.

ICH is a feared complication of BM. In a cohort of 145,225 hospitalized patients with BM, ICH was present in 2.85%, and the two most common underlying primary tumors were melanoma, and kidney cancer [5]. In a prospective series of 2041 patients with intracranial neoplasms and 692 patients with spontaneous ICH, the frequency of ICH in patients with intracranial neoplasms was 2.4%. while 7.2% in the ICH group had tumor-related ICH [6]. In an institutional cohort of 158 patients undergoing evaluation for BM due to renal cell carcinoma (RCC), 94.4% had clear-cell RCC, and 90.6% had extracranial metastases at diagnosis [14]. Our patient also had clear cell RCC and presented with known lung metastases. Patients with a solitary brain lesion (versus multiple) were significantly less likely to develop recurrence, and had a median recurrence-free time of 12.4 months. Many patients with cancer are on anticoagulants due to the increased prevalence of deep venous thrombosis in the population. This poses an additional risk for ICH secondary to metastases [15]. Fortunately, this was not the case with our patient.

## Conclusions

Hemorrhagic BM is not a common etiology for ICH, and initial imaging may not reveal the etiology clearly. It is important to carefully consider the features of the ICH on imaging and obtain further studies if an underlying malignancy is suspected. Even considering the rarity of ICH secondary to BM, it should not be immediately disaffirmed. Improper diagnosis of hemorrhaging relating to metastatic cancer may lead to unnecessary interventions which could further worsen the situation.
